# Association of *IL4R *single-nucleotide polymorphisms with rheumatoid nodules in African Americans with rheumatoid arthritis

**DOI:** 10.1186/ar2994

**Published:** 2010-05-05

**Authors:** Paula I Burgos, Zenoria L Causey, Ashutosh Tamhane, James M Kelley, Elizabeth E Brown, Laura B Hughes, Maria I Danila, Amalia van Everdingen, Doyt L Conn, Beth L Jonas, Leigh F Callahan, Edwin A Smith, Richard D Brasington, Larry W Moreland, Désirée M van der Heijde, Graciela S Alarcón, S Louis Bridges

**Affiliations:** 1 University of Alabama, Tuscaloosa, Alabama 35487, USA; 2 Department of Clinical Immunology and Rheumatology, School of Medicine, Pontificia Universidad Católica de Chile, Avenida Libertador Bernardo O'Higgins 340, Santiago, Chile; 3 Leiden University Medical Center, Albinusdreef 2, 2333 ZA Leiden, The Netherlands; 4 Emory University, 571 Kilgo Circle, Atlanta, GA 30322-1120, USA; 5 University of North Carolina at Chapel Hill, 130 Mason Farm Road, Chapel Hill, NC, USA; 6 Medical University of South Carolina, Jonathan Lucas Street, Charleston, SC29425, USA; 7 Washington University in St. Louis, 4444 Forest Park Avenue, St Louis, MO 63108-2259, USA; 8 Current address: The University of Pittsburgh, Pittsburgh, PA 15260, USA

## Abstract

**Introduction:**

To determine whether *IL4R *single-nucleotide polymorphisms (SNPs) rs1805010 (I50V) and rs1801275 (Q551R), which have been associated with disease severity in rheumatoid arthritis (RA) patients of European ancestry, relate to the presence of rheumatoid nodules and radiographic erosions in African Americans.

**Methods:**

Two *IL4R *SNPs, rs1805010 and rs1801275, were genotyped in 749 patients from the Consortium for Longitudinal Evaluation of African-Americans with Early Rheumatoid Arthritis (CLEAR) registries. End points were rheumatoid nodules defined as present either by physical examination or by chest radiography and radiographic erosions (radiographs of hands/wrists and feet were scored using the modified Sharp/van der Heijde system). Statistical analyses were performed by using logistic regression modeling adjusted for confounding factors.

**Results:**

Of the 749 patients with RA, 156 (20.8%) had rheumatoid nodules, with a mean age of 47.0 years, 84.6% female gender, and median disease duration of 1.9 years. Of the 461 patients with available radiographic data, 185 (40.1%) had erosions (score >0); their mean age was 46.7 years; 83.3% were women; and median disease duration was 1.5 years. Patients positive for *HLA-DRB1 *shared epitope (SE) and autoantibodies (rheumatoid factor (RF) or anti-cyclic citrullinated peptide (CCP)) had a higher risk of developing rheumatoid nodules in the presence of the AA and AG alleles of rs1801275 (odds ratio (OR)_adj _= 8.08 (95% confidence interval (CI): 1.60-40.89), *P *= 0.01 and OR_adj _= 2.97 (95% CI, 1.08 to 8.17), *P *= 0.04, respectively). Likewise, patients positive for the *HLA-DRB1 *SE and RF alone had a higher risk of developing rheumatoid nodules in presence of the AA and AG alleles of rs1801275 (OR_adj _= 8.45 (95% CI, 1.57 to 45.44), *P *= 0.01, and OR_adj _= 3.57 (95% CI, 1.18 to 10.76), *P *= 0.02, respectively) and in the presence of AA allele of rs1805010 (OR_adj _= 4.52 (95% CI, 1.20 to 17.03), *P *= 0.03). No significant association was found between *IL4*R and radiographic erosions or disease susceptibility, although our statistical power was limited by relatively small numbers of cases and controls.

**Conclusions:**

We found that *IL4R *SNPs, rs1801275 and rs1805010, are associated with rheumatoid nodules in autoantibody-positive African-American RA patients with at least one *HLA-DRB1 *allele encoding the SE. These findings highlight the need for analysis of genetic factors associated with clinical RA phenotypes in different racial/ethnic populations.

## Introduction

Rheumatoid arthritis (RA) is a disease of unknown etiology with a prevalence of 1% in populations of European ancestry [1]; it seems to be less prevalent in patients of African ancestry than in persons of European heritage [2]. Predictors of disease severity include environmental factors (socioeconomic status and smoking history), clinical factors (the number of swollen and tender joints and the presence of extraarticular manifestations and erosions at baseline) [3], laboratory data (presence of rheumatoid factor (RF) and/or anticyclic citrullinated peptide antibodies (anti-CCP) [4], higher sedimentation rate (ESR) or C-reactive protein (CRP) levels), and other variables (educational level, score in the Health Assessment Questionnaire (HAQ)). In addition, the *HLA-DRB1 *shared epitope (SE) has been associated with both disease severity and susceptibility [5], and other loci have been associated also with RA in multiple genome-wide association studies (GWASs) [6,7]. Some of these studies have used radiographic parameters to assess disease progression [8,9].

It has been suggested that interleukin-4 (IL-4) and its receptor, encoded by *IL4R*, could play a role in the pathogenesis of RA [8,10] because diminished production of IL-4 may contribute to the T_H_1-mediated autoimmune inflammatory response characteristic of this disease [11,12]. Furthermore, an imbalance in the T_H_1-to-T_H_2 ratio in the adaptive immune response occurs in a variety of immune disorders, including RA. Seven single-nucleotide polymorphisms (SNPs) have been identified in the coding region of *IL4R *[13-15]. Two of these SNPs, rs1805010 (I50V) and rs1801275 (Q551R), are nonsynonymous [16] and are thought to be important in asthma and atopic diseases, as IL-4 plays a major role in IgE production [15,17].

The role of rs1805010 and rs1801275 in RA is controversial. Prot *et al. *[8] reported an association of these genetic variants with the rapid development of erosions and a diminished responsiveness to IL-4 by CD4^+ ^T cells in German RA patients, whereas Marinou *et al. *[18] failed to support such associations in an English population.

Because minority groups are often underrepresented in observational studies and randomized clinical trials [19,20], we evaluated in African-Americans patient with RA whether these *ILR4 *SNPs are associated with rheumatoid nodules and erosions, two clinical manifestations associated with disease severity. This study may improve our understanding of the disease course in these patients and possibly help to identify the presence of ethnic-specific risk factors [19-22].

## Materials and methods

### Study population

The Consortium for the Longitudinal Evaluation of African Americans with Early Rheumatoid Arthritis (*CLEAR*) registry is a National Institute of Arthritis and Musculoskeletal and Skin Diseases-funded program enrolling self-identified African Americans with RA, as defined by the revised American College of Rheumatology (ACR) classification criteria [23]. Patients with disease duration of <2 years were enrolled in *CLEAR I*, a longitudinal cohort, from 2000 to 2005. Patients with any disease duration were enrolled in *CLEAR II*, a cross-sectional arm of the registry, beginning in 2006. Enrollment in *CLEAR II *is ongoing. Comprehensive demographic, clinical, and radiographic data were obtained from *CLEAR I *participants at the baseline visit and at 36 and 60 months from disease onset, and at one time point in *CLEAR II *participants. Data on current and previous drug treatments, including disease-modifying antirheumatic drugs (DMARDs), biologic agents, and glucocorticoids were collected. Participants were recruited, informed consents were obtained from study participants by study coordinators, and studies were conducted under approval by the Institutional Review Boards of the participating institutions: The University of Alabama at Birmingham (UAB) (Coordinating Center), Emory University (Atlanta, GA); The Medical University of South Carolina (Charleston), The University of North Carolina at Chapel Hill, and Washington University (St. Louis, MO). A group of 282 healthy African Americans matched for age and gender without RA, recruited as part of the CLEAR Registry, were used as a control group.

Variables ascertained included age, ethnicity, education, poverty (as defined by the United States Federal Government and adjusted for the number of subjects in the household [24]), history of RA in a first-degree relative, smoking history (self-reported ever or never), disease duration, the ACR core set of variables (number of swollen joints, number of painful joints, pain scale (assessed with a 0 to 10 Visual Analog Scale)) [25], functional status assessed with the Health Assessment Questionnaire [26], and presence of extraarticular manifestation (pulmonary fibrosis, Sjögren syndrome, pericarditis, vasculitis, skin ulcers, or scleritis). The following laboratory variables were recorded at enrollment: serum CRP measured with a high-sensitivity immunometric assay (hsCRP; Immulite 2000 Diagnostic Products, Los Angeles, CA), anti-CCP antibody (IgG) detected with a second-generation enzyme-linked immunosorbent assay (ELISA; Diastat, Axis-Shield Diagnostics, Dundee, Scotland), and rheumatoid factor measured with ELISA (INOVA Diagnostics, San Diego, CA, USA) [27].

### Rheumatoid nodules and radiographic erosions

The presence of rheumatoid nodules was defined by history and physical examination at enrollment. Participants who reported a history of rheumatoid nodules on chest imaging were also considered to have rheumatoid nodules. All RA participants underwent radiographic evaluations of their hands/wrists (posteroanterior views) and feet (anteroposterior views). Study radiographs were read by an experienced reader blinded to all clinical and demographic data, who scored the radiograph for erosions and joint-space narrowing (JSN) by using the van der Heijde modified Sharp method [28,29]. The presence of erosions and JSN for each patient was defined by scores greater than zero; only the baseline evaluations were included in these analyses because of the limited number of follow-up radiographs in this ongoing study [30].

### DNA isolation and genotyping

Genotypes for rs1805010 and rs1801275 were determined by TaqMan (Applied Biosystems (ABI), Foster City, CA) on an ABI 7900 HT Sequence Detection System. *HLA-DRB1 *shared epitope (SE) status was determined as previously described [31].

### Statistical analyses

The analyses were performed by using SAS 9.1 (Cary, NC). Separate comparisons between socioeconomic, demographic, clinical, and laboratory characteristics as a function of the presence of rheumatoid nodules and erosions were performed. Categoric variables were compared by using the Pearson χ^2 ^test, whereas continuous variables were compared with independent *t *tests and the nonparametric Wilcoxon rank-sum test, where appropriate. A two-tailed *P *value of <0.05 was chosen as the statistically significant level. We analyzed potential associations between the *IL4R *SNPs and the presence of rheumatoid nodules and radiographic erosions, by using univariate and multivariate analyses. Logistic regression models were used to analyze the odds of having rheumatoid nodules and erosions as a function of the presence of *IL4R *SNPs, adjusted by nodules or erosions (according to the outcome analyzed), age at RA onset, gender, disease duration (≥2 years versus <2 years), smoking, and methotrexate use. The patients were stratified by the presence of autoantibodies (anti-CCP or RF positive, as one or separate), and *HLA-DRB1 *SE. Hardy-Weinberg equilibrium for the distribution of genotypes was examined with Fisher's Exact test.

## Results

### Patient characteristics

In total, 749 African-American RA patients from CLEAR I and II were analyzed. The mean age at RA onset was 47.0 years, and 84.6% of patients were women. Median disease duration was 1.9 years; 86% of the patients had either anti-CCP antibodies or RF.

Of the 749 RA participants, 156 (20.8%) had rheumatoid nodules; these patients had more comparable ages at disease onset than did those without nodules but were more likely to have longer disease duration, a smoking history, higher HAQ scores, increased presence of joint-space narrowing and of autoantibodies, and higher levels of CRP (Table 1).

**Table 1:** Baseline sociodemographic, clinical, and laboratory characteristics of RA patients in the CLEAR registries as a function of the presence of erosions or rheumatoid nodules

	Radiographic erosions			Rheumatoid nodules		
		
Variable	Present n = 185	Absent n = 276	*P* ^f^	Present n = 156	Absent n = 593	*P* ^f^
Age at RA onset, years, mean (SD)	45.3 (14.5)	48.3 (12.6)	0.02	45.6 (12.6)	47.8 (13.6)	0.06
Disease duration,^a ^years, median (IQR^b^)	3.8 (1.2-15.5)	1.2 (6.0-2.0)	<0.0001	7.5 (1.3-15.6)	1.8 (0.8-8.0)	<0.001
Smoking, ever,^c ^*n *(%)	98 (54.4)	139 (51.9)	0.59	95 (61.7)	295 (50.3)	0.01
HAQ^d ^score, median (IQR)	2.0 (1.3-2.4)	1.8 (0.9-2.4)	0.27	2.1 (1.3-2.6)	1.8 (1.0-2.4)	0.01
Joint-space narrowing at baseline, present (score >0), *n *(%)	132 (71.4)	34 (12.3)	<0.001	48 (53.3)	116 (31.9)	<0.001
Rheumatoid factor positive, *n *(%)	145 (87.3)	182 (74.3)	0.001	127 (90.7)	418 (77.1)	<0.001
Anti-CCP antibody positive, *n *(%)	148 (81.3)	159 (59.3)	<0.001	127 (84.1)	375 (65.2)	<0.001
Abnormal C-reactive protein (>5 mg/L), *n *(%)	94 (51.9)	126 (46.5)	0.26	77 (58.8)	238 (44.7)	<0.004
*HLA-DRB1 *present,^e ^*n *(%)	78 (42.9)	113 (41.1)	0.71	64 (43.2)	234 (39.9)	0.45
Methotrexate use, ever,^c ^*n *(%)	149 (82.3)	175 (64.6)	<0.001	127 (81.9)	423 (72.2)	0.01

^a^Disease duration at enrollment: duration between self-reported onset of symptoms and date of enrollment; ^b^IQR interquartile range (25^th ^and 75^th^); ^c^current or past; ^d^Heath Assessment Questionnaire; ^e^*HLADRB1 *was made to determine SE positive; ^f^Two-tailed *P *value by independent-sample *t *test, Wilcoxon rank-sum test, or χ^2 ^test for proportions, as appropriate and wherever applicable, missing data were excluded from the denominator for calculating percentage and *P *value.

Radiographic scores at enrollment were available in 461 *CLEAR I *and *CLEAR II *RA participants. Of these 461 patients, 185 (40.1%) patients had at least one radiographic erosion. RA participants with erosions were younger at RA onset, had longer disease duration, were more likely to have joint-space narrowing and autoantibodies, and to have been treated with methotrexate (Table 1).

The distribution of patients with other extraarticular manifestations was as follows: 16 (2.2%) pulmonary fibrosis, 21 (2.9%) Sjögren syndrome, six (0.8%) pericarditis, three (0.4%) vasculitis, 11 (1.5%) skin ulcers, and 16 (2.2%) scleritis.

### Lack of association with IL4R genotype and disease susceptibility

Each SNP was in Hardy-Weinberg equilibrium, and the frequencies were similar to those reported in other studies for rs1805010 but different for rs1801275 [8,32,33]. No significant differences were found in the genotype and allele frequencies between controls and RA patients. For rs1805010, frequencies were 23.7% and 26.5% for homozygous AA and GG genotypes, respectively, and 49.8% for the heterozygous AG genotype. Likewise, rs1801275 frequencies were 9.9% and 46.6% for homozygous AA and GG genotypes, respectively, and 43.5% for the heterozygous AG genotype.

### Association of IL4R SNPs with rheumatoid nodules

No significant association between the rs1801275 and rs1805010 alleles and rheumatoid nodules were found in the unadjusted and adjusted analyses (data not shown). After stratification for the presence of *HLA-DRB1 *SE and autoantibody status (RF or anti-CCP) in multivariable models, the rs1801275 AA genotype (OR_adj _= 8.08 (95% CI, 1.60 to 40.89); *P *= 0.01] and AG genotype (OR_adj _= 2.97 (95% CI, 1.08 to 8.17), *P *= 0.04)] were found to be associated with rheumatoid nodules (Table 2). Similarly, stratifying separately by the presence of *HLA-DRB1 *SE and anti-CCP antibodies, the AA and AG genotypes of rs1801275 were associated with rheumatoid nodules (OR_adj _= 8.12 (95% CI, 1.59 to 41.44), *P *= 0.01; and OR_adj _= 2.96 (95% CI, 1.15 to 7.62), *P *= 0.02; respectively) in the multivariable model (data not shown). Likewise, stratifying separately by the presence of *HLA-DRB1 *SE and RF, the rs1801275 AA and AG genotypes (OR_adj _= 8.45 (95% CI, 1.57 to 45.44), *P *= 0.01; and OR_adj _= 3.57 (95% CI, 1.18 to 10.76), *P *= 0.02, respectively] and the rs1805010 AA genotype (OR_adj _= 4.52 (95% CI, 1.20 to 17.03), *P *= 0.03) were associated with rheumatoid nodules (data not shown).

**Table 2:** Association of rs1805010 and rs1801275 with rheumatoid nodules after stratification by the presence of *HLA-DRB1 *SE and autoantibodies in African Americans with RA

**Variable**			**Univariate analysis**		**Multivariate analysis**	
				
	**Rheumatoid nodules *n *(%)**	**No rheumatoid nodules *n *(%)**	**Crude OR (95% CI)**	***P***	**Adjusted^b ^OR (95% CI)**	***P***
	
rs1805010, *n *(%)	n = 51^a^	n = 171^a^				
AA	17 (33.3)	31 (18.1)	1.61 (0.69 to 3.75)	0.27	2.74 (0.80 to 9.41)	0.11
AG	20 (39.2)	99 (57.9)	0.59 (0.27 to 1.28)	0.18	0.64 (0.21 to 1.96)	0.43
GG	14 (27.5)	41 (24.0)	1.00	--	1.00	
						
rs1801275, *n *(%)	n = 51^a^	n = 172^a^				
AA	7 (13.7)	14 (8.1)	2.44 (0.86 to 6.95)	0.09	**8.08 (1.60 to 40.89)**	**0.01**
AG	27 (52.9)	75 (43.6)	1.76 (0.89 to 3.48)	0.11	**2.97 (1.08 to 8.17)**	**0.04**
GG	17 (33.3)	83 (48.3)	1.00	--	1.00	--

^a^Patients with *HLA-DRB1 *SE plus either anti-CCP or RF positive. ^b^Adjusted by age at RA onset (continuous) + Erosion + Gender + Disease duration (≥2 years versus <2 years) + smoking (ever vs. never) + methotrexate (ever versus never).

### Lack of association of IL4R SNPs with radiographic erosions

No significant association between the presence of radiographic erosions and the SNPs examined was found in any of the unadjusted and adjusted analyses performed. Specifically, statistical significance was not reached after stratification by the presence or absence of *HLA-DRB1 *SE and anti-CCP or RF status (alone or combination). These data are shown in Table 3.

**Table 3:** Association of rs1805010 and rs1801275 with radiographic erosions after stratification by the presence of *HLA-DRB1 *SE and autoantibodies in African Americans with RA

**Variable**			**Univariate analysis**		**Multivariate analysis**	
				
	**Erosion^a ^*n *(%)**	**No erosion *n *(%)**	**Crude OR (95% CI)**	***P***	**Adjusted^c ^OR (95% CI)**	***P***
	
rs1805010, *n *(%)	n = 65^b†^	n = 74^b^				
AA	15 (23.1)	15 (20.3)	0.89 (0.34 to 2.36)	0.82	0.95 (0.32 to 2.86)	0.93
AG	31 (47.7)	42 (56.8)	0.66 (0.30 to 1.47)	0.31	0.81 (0.33 to 1.99)	0.65
GG	19 (29.2)	17 (22.9)	1.00	--	1.00	--
						
rs1801275, *n *(%)	n = 65^b^	n = 75^b^				
AA	3 (4.6)	9 (12.0)	0.31 (0.08 to 1.26)	0.10	0.30 (0.06 to 1.47)	0.14
AG	28 (43.1)	34 (45.3)	0.78 (0.39 to 1.55)	0.47	0.64 (0.30 to 1.40)	0.27
GG	34 (52.3)	32 (42.7)	1.00	--	1.00	--

^a^Erosion score >0 at enrollment; ^b^Patients with *HLA-DRB1 *SE, plus either anti-CCP or RF positive; ^c ^Adjusted by age at RA onset (continuous) + Gender + Disease duration (≥2 years versus <2 years) + rheumatoid nodules + smoking (ever versus never) + methotrexate (ever versus never).

## Discussion

This is the first study conducted in African-American RA patients to determine the relation between the occurrence of rheumatoid nodules and erosions and the presence of the nonsynonymous *IL4R *SNPs rs1805010 and rs1801275. We found that the rs1801275 AA and AG genotypes and the rs1805010 AA genotype were associated with rheumatoid nodules in African-American RA patients who are *HLA-DRB1 *SE positive and autoantibody positive.

The minor allele frequency of rs1805010 in African Americans patients with RA was found to be of similar frequency to that in the English and German studies [8,18]; in contrast, the minor allele frequency of rs1801275 was found to be different from that in these two European populations. These findings must be considered to understand the disease phenotypes in patients of different ethnicities.

As expected, rheumatoid nodules were associated with disease duration, smoking, HAQ score, JSN, RF, anti-CCP antibodies, elevated CRP levels, and methotrexate use. However, *HLA-DRB1 *SE status alone does not appear to increase significantly the risk of rheumatoid nodules among African Americans. Several studies conducted in patients of European descent found no association between the SE and rheumatoid nodules [34-37], whereas others found such an association [38,39]. Thus, the genetic predisposition to the development of rheumatoid nodules is complex and involves more than the presence of the SE and its interaction with environmental factors, such as smoking [40,41] or medications [42]. However, the SE has a smaller absolute effect in African Americans than in individuals of European descent [20,31].

The presence of radiographic erosions was associated with age, disease duration, the presence of JSN, RF, anti-CCP antibodies, and methotrexate use, but it was not associated with *HLA-DRB1 *SE, CRP levels, HAQ score, or smoking. After stratification by the presence of the SE and RF or anti-CCP antibodies (alone or in combination), no association between erosions and rs1805010 or rs1801275 was found. Similar data were reported by Marinou *et al. *[18] in a cross-sectional study of 965 white RA patients and 988 healthy controls; however, our data do not corroborate the report by Prots *et al. *[8] of a higher risk of developing early erosions, as noted in their longitudinal German cohort of 302 RA patients. Perhaps this effect was not observed because of the different racial/ethnic composition of the patients studied; in African Americans, these polymorphisms could be modulators of other phenotypes.

Because RF and anti-CCP antibodies have similar sensitivities, but anti-CCP antibodies have a higher specificity [27], we decided to stratify by these autoantibodies when assessing the relation with rheumatoid nodules and radiographic erosions. We found no association between *HLA-DRB1 *SE/anti-CCP positivity with rheumatoid nodules or erosions, but a new association was found between rs1805010 (AA genotype) and *HLA-DRB1 *SE/RF positivity. Thus, *HLA-DRB1 *SE/RF-positive patients with the AA genotype of rs1805010 and the AA or AG genotype of rs1801275 have a higher risk of rheumatoid nodules than do those patients without this feature. The higher sensitivity and specificity of RF for the presence of extraarticular manifestations as compared with anti-CCP antibodies, as reported in other studies [4,43,44], is a probable explanation for our findings.

There are several limitations to our study. Complete radiographic baseline data and genotyping are not available because the study is ongoing. Only 25% of *CLEAR I *RA participants had erosions at baseline, given the enrollment criterion of disease onset <2 years. In addition to restricting the analysis to a small number of subjects, the inclusion of early RA patients might miss a subset of patients who would go on to develop extraarticular manifestations with longer disease duration. Furthermore, we only analyzed two SNPs of *ILR4*, and our results could be influenced by the interactions of other SNPs within these genes or other genes whose proteins are associated with T_H_1-to-T_H_2 balance.

## Conclusions

In conclusion, we found that both rs1801275 and rs1805010 are associated with rheumatoid nodules in African-American RA patients with the *HLA-DRB1 *SE and autoantibodies. These findings may allow future stratification of the risk of extraarticular manifestations in RA.

## Abbreviations

ACR: American College of Rheumatology; CCP: anti-cyclic citrullinated peptide antibodies; *CLEAR*: The Consortium for the Longitudinal Evaluation of African Americans with Early Rheumatoid Arthritis; CRP: C-reactive protein; DMARDs: disease-modifying antirheumatic drugs; ESR: erythrocyte sedimentation rate; GWASs: multiple genome-wide association studies; HAQ: Heath Assessment Questionnaire; IL-4: interleukin-4; JSN: joint-space narrowing; RA: rheumatoid arthritis; RF: rheumatoid factor; SE: shared epitope; SNPs: single-nucleotide polymorphisms.

## Competing interests

The authors declare that they have no competing interests.

## Authors' contributions

LBH, MID, DLC, BLJ, LFC, EAS, RB, LWM, GSA, and SLB investigators participated in the CLEAR study conception, recruiting of patients, and data acquisition. AVE and DVH performed radiographic evaluations. PIB, ZLC, AT, EEB, GSA, and LB participated in the design of the IL4R study. PIB, ZLC, and GSA wrote the initial draft of the manuscript. AT performed the statistical analyses. AT, PIB, ZLC, JMK, and EEB helped with the interpretation. All the authors helped with the final manuscript. All authors read and approved the final manuscript.
